# Lipid Alterations in African American Men with Prostate Cancer

**DOI:** 10.3390/metabo12010008

**Published:** 2021-12-22

**Authors:** Anindita Ravindran, Danthasinghe Waduge Badrajee Piyarathna, Jie Gohlke, Vasanta Putluri, Tanu Soni, Stacy Lloyd, Patricia Castro, Subramaniam Pennathur, Jeffrey A. Jones, Michael Ittmann, Nagireddy Putluri, George Michailidis, Thekkelnaycke M. Rajendiran, Arun Sreekumar

**Affiliations:** 1 Department of Molecular and Cellular Biology, Baylor College of Medicine, One Baylor Plaza, Houston, TX 77030, USA; anindita.ravindran@bcm.edu (A.R.); DanthasingheWaduge.Piyarathna@bcm.edu (D.W.B.P.); jie.gohlke@bcm.edu (J.G.); stacy.lloyd@bcm.edu (S.L.); putluri@bcm.edu (N.P.); 2 Center for Metabolism and Experimental Therapeutics, Baylor College of Medicine, One Baylor Plaza, Houston, TX 77030, USA; 3 Advanced Technology Core, Baylor College of Medicine, One Baylor Plaza, Houston, TX 77030, USA; vputluri@bcm.edu; 4 Michigan Regional Comprehensive Metabolomics Resource Core, University of Michigan, Ann Arbor, MI 48105, USA; tanusoni@med.umich.edu (T.S.); tmraj@med.umich.edu (T.M.R.); 5 Dan L Duncan Comprehensive Cancer Center, Baylor College of Medicine, One Baylor Plaza, Houston, TX 77030, USA; jajones@bcm.edu (J.A.J.); mittmann@bcm.edu (M.I.); 6 Department of Pathology and Immunology, Baylor College of Medicine, One Baylor Plaza, Houston, TX 77030, USA; pcastro@bcm.edu; 7 Human Tissue Acquisition & Pathology Shared Resource, Dan L Duncan Comprehensive Cancer Center, Baylor College of Medicine, Houston, TX 77030, USA; 8 Division of Nephrology, Department of Medicine, University of Michigan, Ann Arbor, MI 48105, USA; spennath@med.umich.edu; 9 Department of Molecular and Integrative Physiology, University of Michigan, Ann Arbor, MI 84105, USA; 10 Department of Urology, Baylor College of Medicine, One Baylor Plaza, Houston, TX 77030, USA; 11 Operative Care Line, Urology Section, Michael E. DeBakey Veterans Affairs Medical Center, Houston, TX 77030, USA; 12 Informatics Institute, University of Florida, Gainesville, FL 32611, USA; gmichail@ufl.edu; 13 Department of Pathology, University of Michigan, Ann Arbor, MI 48105, USA; 14 Michigan Center for Translational Pathology, University of Michigan, Ann Arbor, MI 48105, USA

**Keywords:** lipidomics, prostate cancer health disparity, biochemical recurrence, cholesteryl esters

## Abstract

African-American (AA) men are more than twice as likely to die of prostate cancer (PCa) than European American (EA) men. Previous in silico analysis revealed enrichment of altered lipid metabolic pathways in pan-cancer AA tumors. Here, we performed global unbiased lipidomics profiling on 48 matched localized PCa and benign adjacent tissues (30 AA, 24 ancestry-verified, and 18 EA, 8 ancestry verified) and quantified 429 lipids belonging to 14 lipid classes. Significant alterations in long chain polyunsaturated lipids were observed between PCa and benign adjacent tissues, low and high Gleason tumors, as well as associated with early biochemical recurrence, both in the entire cohort, and within AA patients. Alterations in cholesteryl esters, and phosphatidyl inositol classes of lipids delineated AA and EA PCa, while the levels of lipids belonging to triglycerides, phosphatidyl glycerol, phosphatidyl choline, phosphatidic acid, and cholesteryl esters distinguished AA and EA PCa patients with biochemical recurrence. These first-in-field results implicate lipid alterations as biological factors for prostate cancer disparities.

## 1. Introduction

Prostate cancer (PCa) progression is twice as aggressive and more than twice as morbid in African American (AA) men compared to European American (EA) men, yet the specific biochemical basis underlying this disparity remains unknown [1]. While environmental and socioeconomic factors such as diet and access to proper healthcare may be significant causes for increased mortality rates associated with PCa in AA men, recent reports suggest that metabolic alterations may also drive the disparity in PCa progression [2]. Our previous in silico study described enriched adipogenesis, fatty acid (FA) metabolism, and cholesterol homeostasis, among others, as being enriched in AA tumors across multiple different tumor types, which was further validated in an independent PCa gene expression dataset [3]. Earlier work performed on fatty acid (FA) metabolism in AA men revealed higher levels of arachidonic acid (a long chain polyunsaturated FA (PUFA) implicated in inflammation) and single nucleotide polymorphisms (SNPs) in the fatty acid desaturase (FADS) locus [4]. In addition, elevated levels of triglycerides in plasma have been associated with PCa risk and poor clinical outcome in AA men [5]. Similarly, elevated cholesterol levels in serum have been reported to be associated with increased biochemical recurrence (BCR), specifically in AA men with PCa [5]. However, the global changes in the lipidome in AA and EA PCa tissues have not been examined.

We, therefore, sought to examine the global lipidome changes in prostate tissues of AA and EA PCa patients, to identify alterations in lipid metabolism that contribute to the observed disparity in tumor progression. Patient tissues were stratified by Gleason score into low (Gleason ≤ 6 and Gleason 3 + 4) or high (Gleason >7 or Gleason (4 + 3)) groups. In addition, patients were stratified based on biochemical recurrence (BCR), a clinical endpoint that is defined by prostate-specific antigen (PSA) levels ≥0.2 ng/mL above the nadir, following radical prostatectomy or radiation. Overall, in this study, we highlight alterations in classes of lipids associated with PCa in a race-independent and dependent manner. In addition, changes in specific lipids associated with low vs. high Gleason grade, and BCR are described.

## 2. Results

We performed unbiased global lipidomic profiling using triple time of flight (Triple-TOF) liquid chromatography-mass spectrometry (LC-MS) on 48 pathologically-verified frozen localized PCa and matched benign adjacent tissue (Table 1 for Clinical Information, 30 AA and 18 EA pairs, 24 AA and 8 EA tissues were ancestry verified). The profiling platform was highly reproducible, with an average coefficient of variation (CV) of 2.7% in the liver pool (*n* = 24) for 429 lipids measured, an average CV of 5.3% for internal standards (*n* = 26 spiked into clinical samples, Supplementary Figure S1), and normal distribution of the lipids overall and within each class (Supplementary Figure S2). The 429 lipid species identified belonged to 14 lipid classes (See Table 2 for the number of lipids measured in each class), including cholesteryl esters (CE), diacylglycerols (DG), lysophosphatidyl cholines (L-PC), lysophosphatidyl ethanolamines (L-PE), phosphatidic acid (PA), phosphatidyl cholines (PC), phosphatidyl ethanolamines (PE), phosphatidyl glycerol (PG), phosphatidyl inositols (PI), plasmenyl-phosphatidyl cholines (P-PC), plasmenyl-phosphatidyl ethanolamines (P-PE), phosphatidyl serines (PS), sphingomyelins (SM), and triglycerides (TG).

**Figure 1:**
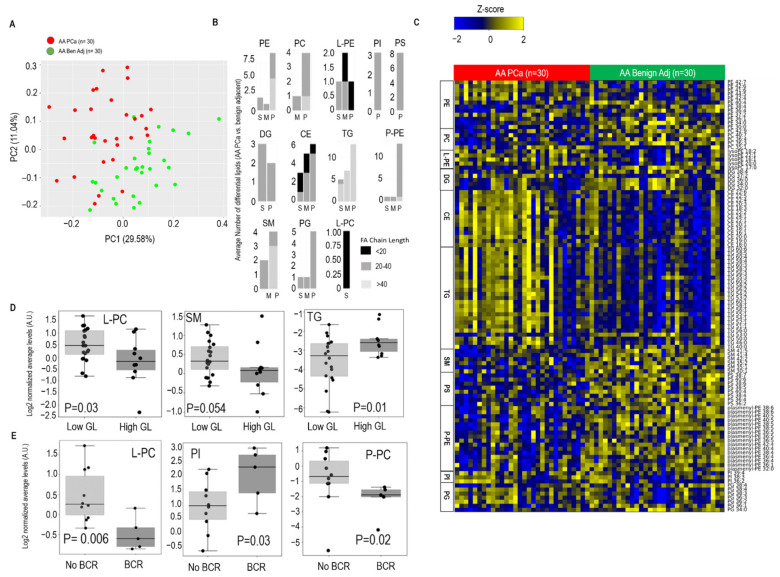
Altered lipidome in AA PCa vs. matched benign adjacent prostate tissue. (**A**) Principal component analysis using lipid profiles in 30 paired AA PCa and benign adjacent prostate tissues. (**B**) Number of altered lipids within each class stratified by fatty acid chain length (see legend) and degree of saturation. S: saturated, M: mono-unsaturated, P: poly-unsaturated (≥2 double bonds). (**C**) Heat map showing significantly altered lipids (stratified by fatty acid chain length: degree of saturation) in AA PCa vs. matched benign patient prostate tissues. Shades of yellow and blue represent up and down regulated lipids, respectively (see color key). Lipid classes are marked on the left side of the heatmap. Lipid classes include PE: Phosphatidyl ethanolamine, PC: Phosphatidyl choline, PI: Phosphatidyl inositol, PG: Phosphatidyl Glycerol, PS: Phosphatidyl Serine, L-PE: Lyso Phosphatidyl Ethanolamine, TG: Triglycerides, SM: Sphingomyelin, P-PE: Plasmenyl Phosphatidyl Ethanolamine, CE: Cholesteryl Esters, DG: Diglycerides, L-PC: Lyso phosphatidyl choline. (**D**) Average levels of L-PC (*p* = 0.03) and SM (*p* = 0.054) are significantly down-regulated, and average levels of TGs (*p* = 0.01) are significantly elevated in high Gleason grade (High GL, *n* = 10) compared to low Gleason grade (Low GL, *n* = 20) tumors. (**E**) Average lower levels of L-PC (*p* = 0.006) and P-PC (plasmenyl-phosphatidyl choline, *p* = 0.02) and elevated average levels of PI (*p* = 0.03) are associated with biochemical recurrence (BCR, 5 years post-prostatectomy) in PCa patients. No BCR (*n* = 10); BCR (*n* = 5). For panels B and C, paired *t*-test followed by Benjamini Hochberg (BH) false discovery rate (FDR < 0.1) correction was used to compute differential analysis. For panels D and E, a Mann–Whitney test with BH FDR < 0.25 was used to compute statistical significance.

Principal component analysis using all the lipids revealed a moderate separation of the benign adjacent tissue and PCa, both across the entire cohort (Supplementary Figure S3A, refer Supplementary Table S1 list of lipids in PC1 and PC2) and within AA patients (Figure 1A, refer Supplementary Table S2 list of lipids in PC1 and PC2), suggestive of alterations in lipid components between these two tissue types. Similarly, across the entire cohort (Supplementary Figure S3B), as well as within AA patients (Figure 1B), comparing PCa with benign adjacent tissue, the significantly altered lipids (FDR < 0.1) were categorized into saturated, monounsaturated, and polyunsaturated groups, having fatty acid chains ranging from <20 to >40 fatty acid residues. Notably, the majority of the altered lipids across all classes were polyunsaturated (>2 double bonds) with long chain fatty acid chains (≥20, Supplementary Figures S1B and S3B). Furthermore, comparing PCa and adjacent benign tissue across all patients (Supplementary Figure S3C, FDR < 0.1), as well as within AA patients (Figure 1C, FDR < 0.1), prominent elevations were seen in the levels of individual lipids belonging to CE and TG. In contrast, the levels of individual lipids belonging to SM, PS, P-PE, PI, and PG were reduced in both comparisons (refer Supplementary Tables S3 and S4 for list of altered lipids). Furthermore, both, across all patients and within AA patients, averaging the samples over lipid classes revealed a prominent elevation in levels of CE, TG, and L-PE and reduced levels of P-PE and PS, in the PCa vs. benign adjacent tissue comparison (Supplementary Figure S4A–H,J,K, FDR < 0.25). In addition, across all patients, the levels of P-PC and PA were also reduced in the PCa vs. benign adjacent tissue comparison (Supplementary Figure S4I,L, FDR < 0.25).

We next looked for altered lipids within PCa that could distinguish low vs. high Gleason grade tumors, as well as identify patients who had biochemical recurrence (BCR) within 5 years post-surgery, a clinical indicator of aggressive PCa. Interestingly, reduced levels of L-PC were associated with high Gleason tumors (Gleason > 7 or 4 + 3, Supplementary Figure S3D, FDR < 0.25) across all PCa patients. Furthermore, within AA PCa, reduced levels of PC and SM and elevated levels of TG were associated with high Gleason tumors (Figure 1D, FDR < 0.25). In addition, across all patients, lower levels of L-PC, SM, and PE were significantly associated with early BCR (FDR < 0.25, Supplementary Figure S3D). Along similar lines, reduced levels of L-PC and P-PC, and elevated levels of PI, were associated with early BCR within AA PCa patients (FDR < 0.25, Figure 1E).

**Figure 2:**
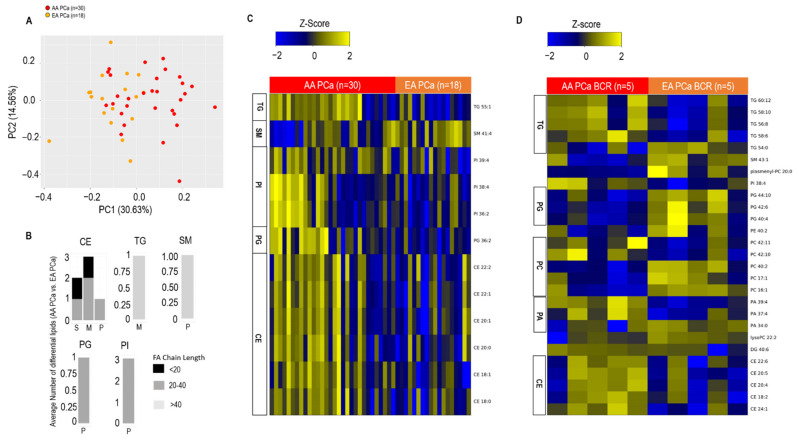
Altered lipidome in AA PCa vs. EA PCa tissue. (**A**) Principal component analysis using lipid profiles in 30 AA and 18 EA PCa tissues. (**B**) Number of altered lipids within each class stratified by fatty acid chain length (see legend) and degree of saturation. S: saturated, M: mono-unsaturated, P: poly-unsaturated (≥2 double bonds). (**C**) Heat map showing significantly altered lipids (stratified by fatty acid chain length: degree of saturation) in AA PCa vs. EA PCa patient prostate tissues. Shades of yellow and blue represent up and down regulated lipids, respectively (see color key). Lipid classes are marked on the left side of the heatmap. Lipid classes include TG: Triglycerides, SM: Sphingomyelin, PI: Phosphatidyl inositol, PG: Phosphatidyl Glycerol, CE: Cholesteryl Esters. (**D**) Heat map showing significantly altered lipids (stratified by fatty acid chain length: degree of saturation) comparing AA PCa and EA PCa with biochemical recurrence (BCR) within 5 years post-prostatectomy. Shades of yellow and blue represent up and down regulated lipids, respectively (see color key). Lipid classes are marked on the left side of the heatmap. For panels (**B)** and (**C)**, a permutation *t*-test coupled to Benjamin Hochberg false discovery rate (FDR < 0.1) correction was used to compute differential analysis. For panel (**D)**, a Mann–Whitney test coupled to Benjamin Hochberg false discovery rate (FDR < 0.35) correction was used to compute differential analysis. For panel (**D)**, altered lipids were selected at a minimum fold change of 2 with *p*-value < 0.05 (FDR < 0.35), comparing AA PCa BCR vs. EA PCa BCR.

Intriguingly, there were key changes in lipid profiles between clinically comparable AA (*n* = 30) and EA (*n* = 18) tumors. As shown in Figure 2A, principal component analysis (refer Supplementary Table S5 for list of lipids in PC1 and PC2) of the lipid profiles was able to delineate AA and EA tumors. Furthermore, lipids (predominantly polyunsaturated and long chain) belonging to 10 lipid classes significantly distinguished AA and EA PCa (Figure 2B). This included prominent elevations (FDR < 0.1) in the level of individual lipids belonging to the CE, TG, PI, and PG class of lipids (Figure 2C, refer Supplementary Table S6 for list of altered lipids) when comparing AA vs. EA PCa. Furthermore, when comparing AA vs. EA PCa, averaging the samples over lipid classes revealed a significant elevation (FDR < 0.25) in levels of CE and PI classes of lipids (Supplementary Figure S4M,N). Interestingly, we also observed at least a two-fold elevation (FDR < 0.35) in levels of individual lipids belonging to the TG, CE, and PI classes in AA tumors with early BCR compared to corresponding EA tumors (Figure 2D). In contrast, using the same criteria, individual lipids belonging to SM, P-PC, and PG were reduced in the same comparison. This, for the first time, demonstrates a race-specific deregulation of lipid pathways in aggressive tumors.

## 3. Discussion

Our findings, for the first time, describe unique race-associated alterations in specific lipid classes when comparing PCa and benign adjacent tissue (diagnostic comparison), as well as within subsets of patients who had undergone BCR (prognostic comparison). Among the classes of lipids, CE is most prominently altered across both diagnostic and prognostic comparisons.

CE is the primary source of cholesterol for steroidogenesis [6]. CE (specifically cholesterol oleate) was detected in previous lipidomics screens and proposed as a potential biomarker for detection of PCa [7]. Excess free cholesterol is esterified and stored as CE in lipid droplets, which is then mobilized by lecithin-cholesterol acyltransferase (LCAT). Notably, LCAT transcript is elevated in AA tumors compared to EA PCa in the The Cancer Genome Atlas (TCGA) dataset. An increase in CE was also shown to be associated with PCa tumor progression and metastasis, and aberrant accumulation of CE was found to be correlated with PTEN loss and PI3K/Akt activation [8,9]. A PIK3CD-S splice variant recently identified in AA PCa patients was shown to promote proliferative signaling [10], suggesting a key role for PI3K/Akt signaling in the development of AA PCa. This is consistent with our previous publication, where we reported that the oncogene MNX1 is upregulated in AA men in response to activated PI3K/AKT signaling [11]. In line with this, in our dataset, we observed a significant elevation in the levels of an important PI3K/Akt pathway second messenger, PI, in AA vs. EA PCa comparison.

In addition, the high levels of TG seen in AA PCa are known to be associated with PCa progression [12]. TG is an important energy source when hydrolyzed into free fatty acids (FFAs). Cancer cells use the energy generated from these TG pools to drive tumor proliferation, specifically under hypoxia [13]. Intriguingly, the increase in individual lipids belonging to CE and TG, in AA vs. EA patients with early BCR, is a novel finding. Elevated levels of these lipids could be regulated by androgens, which are known to affect TG and CE pools by contributing to lipid synthesis [14].

Interestingly, in our study, the majority of the altered lipids in PCa in general, as well as within AA patients, include polyunsaturated fatty acids (PUFAs). PUFAs are known to be high in the AA diet, which predominantly comprises corn and soy [15]. Consistent with this, as mentioned earlier, compared to EA, AA men with PCa have significantly higher circulating levels of arachidonic acid [4]. Our finding that the AA PCa lipidome is rich in long chain PUFAs further supports these prior observations and potentially reveals an impact of diet on PCa risk or progression.

The high incidence of PCa, as well as the aggressive nature of tumor progression in AA men, make it imperative to seek novel biological means to comprehend the disparity. Understanding the alterations in lipid composition, and thereby discovering lipid biomarkers that are prognostic for PCa progression in AA but not EA men, may be critical for developing race-associated therapeutic strategies. Our finding of increased lipids belonging to CE and TG classes in AA tumors, as well as within subsets of AA PCa patients with early BCR, suggests a prognostic value for these lipids in the context of AA PCa.

Taken together, our descriptive data for the first time reveal key changes in the PCa lipidome in AA compared to EA PCa patients, a subset of which are associated with Gleason grade and early BCR.

## 4. Materials and Methods

Frozen pathologically-verified prostate tissues (PCa and adjacent benign) for lipidomics profiling were obtained from the Baylor College of Medicine Human Tissue Acquisition and Pathology Core of the Dan L. Duncan Cancer Center. All samples were collected during radical prostatectomy, with informed consent and Institutional Review Board approval, and stored retrospectively in a de-identified manner in the tumor bank. All tissue samples were reviewed for their tumor content by a genitourinary (GU) pathologist prior to the analysis. Tissue obtained from the cancer samples for lipidomics analysis contained at least 70% tumors, while benign adjacent tissues were free of tumors upon pathological examination.

Lipid extraction from tissues and mass spectrometry analysis were performed as described previously [16,17,18,19]. Briefly, a modified Bligh–Dyer method [20] was used to extract the lipids. Briefly, the extraction was performed using a 2:2:2 volume ratio of water/methanol/dichloromethane at room temperature post spiking with lipid class-specific internal standards (*n* = 26). The organic layer was collected, dried under liquid nitrogen, and resuspended in 100 μL of Buffer B (10:5:85 Acetonitrile/water/Isopropyl alcohol) containing 10 mM Ammonium Acetate. LC/MS with reverse-phase chromatography was used to separate the lipids [19].

A Shimadzu CTO-20A Nexera X2 UHPLC systems with a degasser, binary pump, temperature controlled autosampler, and a column oven for chromatographic separation was used. Lipids were separated by injecting 5 uL of lipid extract through a 1.8 μm particle 50 × 2.1 mm Acquity HSS UPLC T3 column (Waters, Milford, MA, USA). Data acquisition was performed in both positive and negative ionization modes using a TripleTOF 5600 equipped with a Turbo VTM ion source (AB Sciex, Concord, ON, Canada). To acquire the MS2 spectra, we used the data-dependent acquisition (DDA) function of the Analyst TF software (AB Sciex, Concord, ON, Canada), which includes dynamic exclusion to avoid multiplying charged ions and isotopes and to obtain deeper MS2 coverage.

Missing values in the data were imputed using the K nearest-neighbor method (KNN). Data was log2 transformed, day median normalized, and checked to ensure normality, both with in each lipid class and across all lipids measured (Supplementary Figure S2).

For Figure 1B,C (comparing AA PCa vs. benign adjacent tissue), and Supplementary Figure S3B,C (comparing PCa vs. benign adjacent across all patients), differential lipids were determined by paired *t*-test (*p* < 0.05), followed by the Benjamini–Hochberg (BH) procedure for false discovery rate correction (FDR < 0.1). BH was used to control the probability of a Type I error rate due to the testing of multiple hypotheses. For Figure 1D,E, as well as for Supplementary Figure S3D, the FDR value was set at <0.25 (for low vs. high Gleason, and for No BCR vs. BCR comparisons, both within AA PCa patients, and across all PCa patients). Specifically, in the analysis comparing Gleason grade and BCR (Figure 1D,E, and Supplementary Figure S3D), normalized data for all lipids in each class were independently averaged to obtain an average score for the lipid class. Following this, the class-specific scores were compared between the groups using a Mann–Whitney test and corrected for FDR <0.25. A similar approach was used to create Supplementary Figure S4, describing class-specific lipid changes for PCa vs. benign adjacent, AA PCa vs. benign adjacent, and AA vs. EA PCa (FDR < 0.25).

For Figure 2B,C, comparing AA vs. EA PCa, to begin with, AA and EA PCa-associated lipid profiles were obtained by subtracting their respective paired benign values. Following this, the differential lipids between AA and EA PCa were computed using permutation *t*-test (*p* < 0.05) followed by the Benjamini–Hochberg (BH) procedure for false discovery rate correction (FDR < 0.1). For Figure 2D, a Mann–Whitney test coupled to Benjamin Hochberg false discovery rate (FDR < 0.35) correction was used to compare AA PCa BCR vs. EA PCa BCR.

The normalized data for this study have been deposited to the Metabolomics Workbench (https://www.metabolomicsworkbench.org/, accessed on 19 December 2021) under the studyID 001971.

## Figures and Tables

**Table 1:** Clinical parameters of prostate tumor samples used for lipidomics profiling. ŷ * represents the estimated value of the response variable.

Variable	African American (*n* = 30)	European American (*n* = 18)
Gleason Grade	Low (≤6 and 7 = 3 + 4): 20	Low (≤6 and 7 = 3 + 4): 6
	High (>7 and 7 = 4 + 3): 10	High (>7 and 7 = 4 + 3): 12
Recurrence (BCR)	5	5
No Recurrence	10	5
West African, ŷ *	0.8 ± 0.1	0.02 ± 0.02
European, ŷ *	0.2 ± 0.1	0.02 ± 0.02
Native American, ŷ *	0.0 ± 0.0	0.95 ± 0.03
Genetic Ancestry Verified (n)	24	8

**Table 2:** List of lipids detected in each lipid class by unbiased mass spectrometry analysis.

Lipid Class	Number of Lipids Measured
Cholesteryl Ester (CE)	17
Diglycerides (DG)	36
Lyso-Phosphatidyl Choline (L-PC)	11
Lyso-Phosphatidyl Ethanolamine (L-PE)	18
Phosphatidic Acid (PA)	7
Phosphatidyl Choline (PC)	75
Phosphatidyl Ethanolamine (PE)	55
Phosphatidyl Glycerol (PG)	28
Phosphatidyl Inositol (PI)	6
Plasmenyl Phosphatidyl Choline (P-PC)	2
Plasmenyl Phosphatidyl Ethanolamine (P-PE)	21
Phosphatidyl Serine (PS)	21
Sphingomyelin (SM)	34
Triglycerides (TG)	90
Unknown	8
Total	429

## Data Availability

The normalized data for this study have been deposited to the Metabolomics Workbench (https://www.metabolomicsworkbench.org/ accessed on 19 December 2021) under the studyID 001971.

## Supplementary Materials

The following supporting information can be downloaded at: https://www.mdpi.com/article/10.3390/metabo12010008/s1, Figure S1: Quality control data for the lipidomics profiling mass spectrometry platform, Figure S2: Quantile-quantile plots for all lipid classes and individual lipid classes, Figure S3: Altered lipidome in Prostate Cancer (PCa) vs. matched benign adjacent tissue, Figure S4: Box plots for altered lipid classes; Table S1: List of lipids that were loaded onto axes on Principal Component Analysis plots for AA PCa vs. matched benign tissues, Table S2: List of lipids that were loaded onto axes on Principal Component Analysis plots for total PCa vs. matched benign tissues, Table S3: List of differential lipids (FDR < 0.10) in AA PCa vs. matched benign adjacent tissues, Table S4: List of differential lipids (FDR < 0.10) in PCa vs. adjacent benign tissues, Table S5: List of lipids that were loaded onto axes on Principal Component Analysis plots for AA PCa vs. EA PCa tissues, Table S6: List of differential lipids (FDR < 0.10) comparing AA vs. EA PCa tumors.
