# Supplementary material for: Lipid Alterations in African American Men with Prostate Cancer
Source: Metabolites. 2021 Dec 22;12(1):8. doi: 10.3390/metabo12010008 (PMC8779756; doi:10.3390/metabo12010008)
Supplement: Supplementary file 1 [file metabolites-12-00008-s001.zip › metabolites-1471570-supplementary/Supplementary Figures.pdf]

Lipid Alterations in African American Prostate Cancer

Anindita Ravindran[1‡], Danthasinghe Waduge Badrajee Piyarathna[1‡], Jie Gohlke[1‡], Vasanta Putluri[2], Tanu Soni[3], Stacy Lloyd[1,4], Patricia Castro[5,10], Subramaniam Pennathur[6], Jeffrey A Jones[4,7], Michael Ittmann[4.5], Nagireddy Putluri[1,2,4], George Michailidis[8#], Thekkelnaycke M Rajendiran[3,9,#], Arun Sreekumar[1,4,#,*]

Supplementary Figures

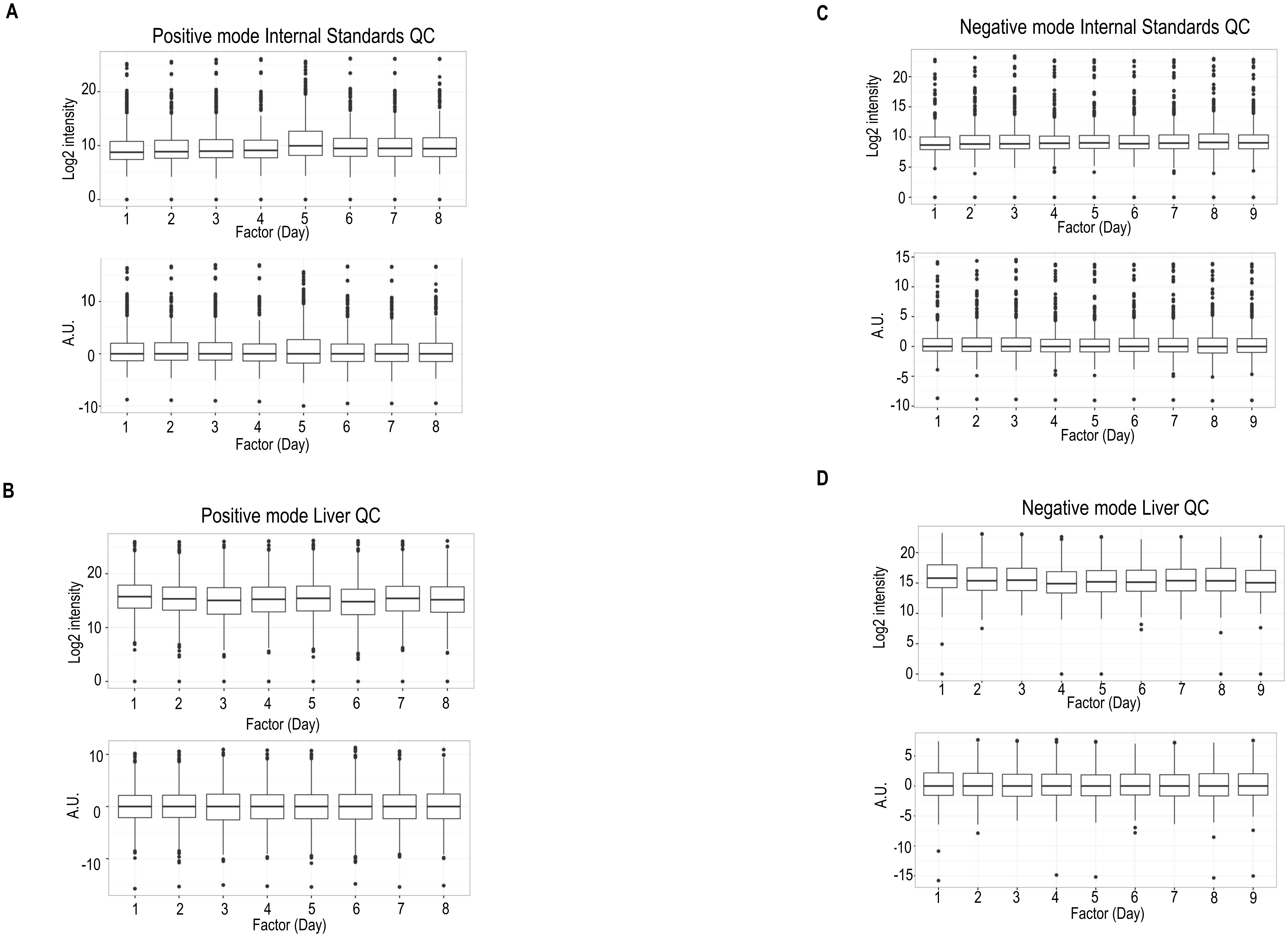

Supplementary Figure S1. Quality control data for the lipidomics profiling mass spectrometry platform. **(A)** and **(B)** show distribution of internal standards for positive and negative ionization modes. Lower panel shows the raw data in arbitrary units (AU) across the nine days of analysis. The upper panel shows the normalized data in log2 scale. **(C)** and **(D)** similar to (A) and (B), but for liver pools. Three liver pool samples were run per day across eight days.

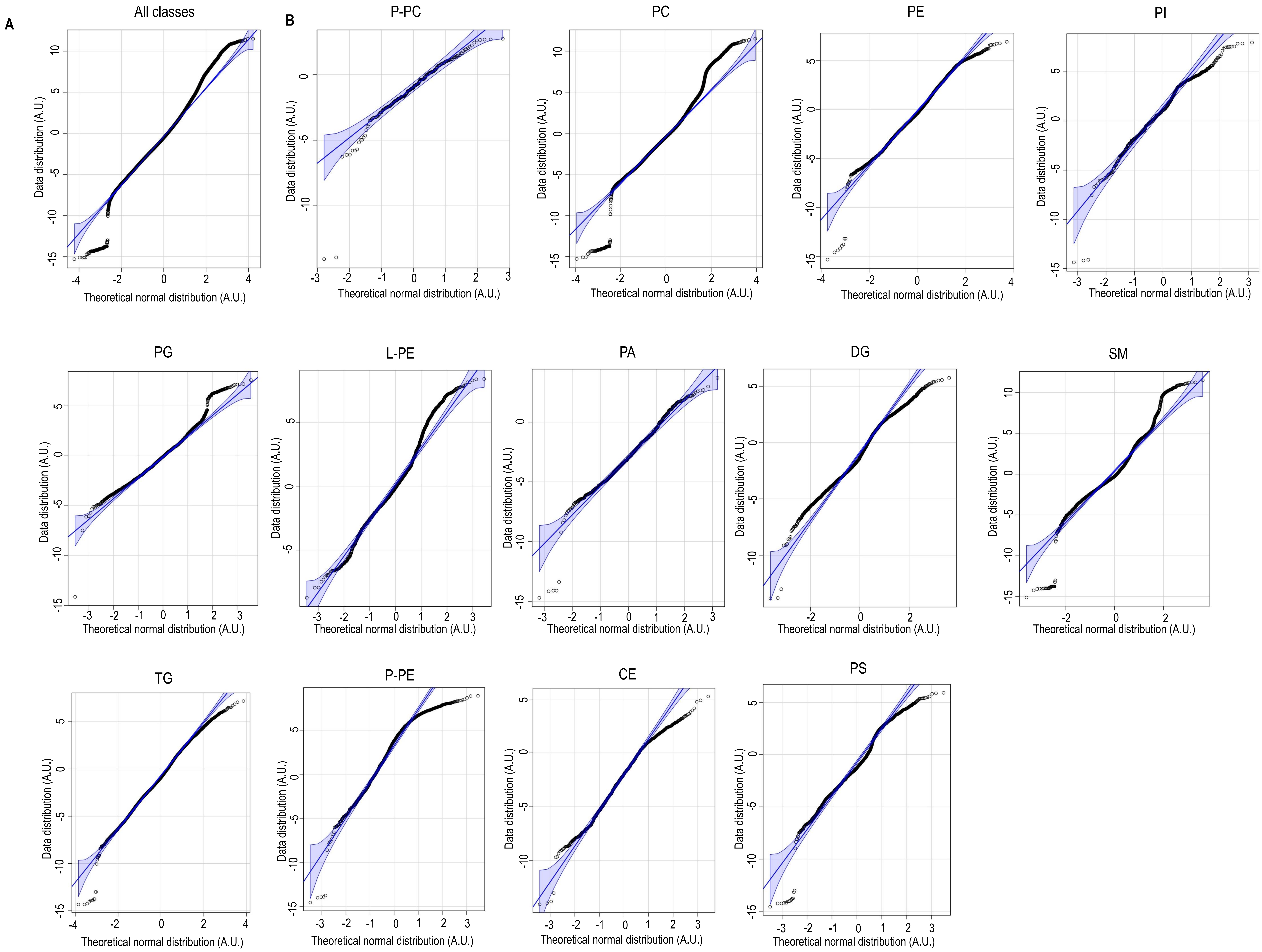

Supplementary Figure S2. Quantile-quantile plots for **(A)** All lipid classes and **(B)** individual lipid classes. P-PC: Plasmenyl-Phosphatidyl Choline, PE: Phosphatidyl ethanolamine, PC: Phosphatidyl choline, PI: Phosphatidyl inositol, PG: Phosphatidyl Glycerol, PS: Phosphatidyl Serine, L-PE: Lyso-Phosphatidyl Ethanolamine, TG: Triglycerides, SM: Sphingomyelin, P-PE: Plasmenyl-Phosphatidyl Ethanolamine, CE: Cholesteryl Esters, DG: Diglycerides, L-PC: Lyso-Phosphatidyl Choline, PA: Phosphatidic Acid.

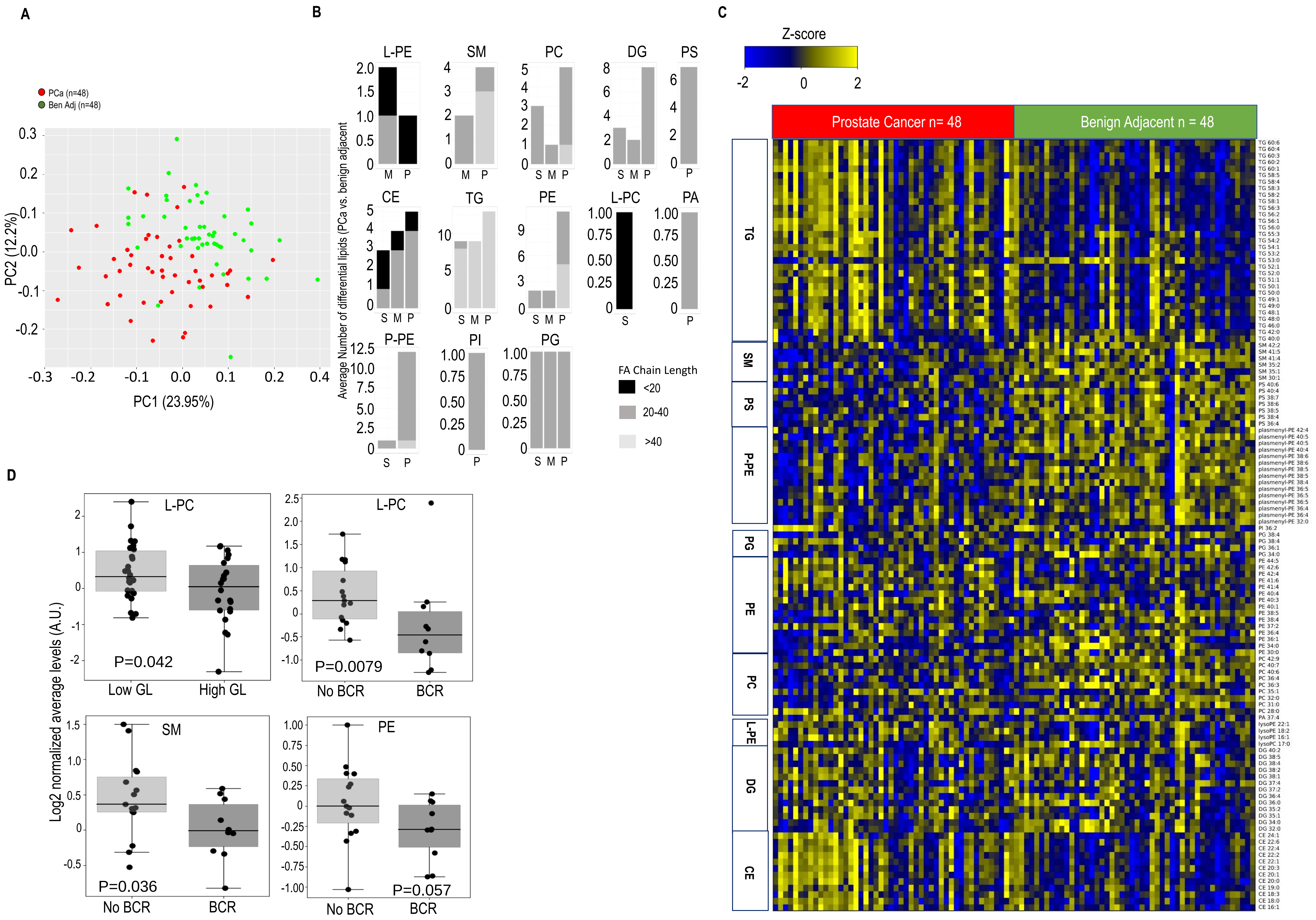

Supplemental Figure S3. Altered lipidome in Prostate Cancer (PCa) vs matched benign adjacent tissue. (A) PCA plot using lipid profiles in 48 paired patient-derived PCa and benign adjacent tissues. **(B)** Number of altered lipids within each class stratified by fatty acid chain length (see legend) and degree of saturation. S: Saturated, M: Mono-unsaturated, P: Poly-unsaturated (≥ 2 double bonds). **(C)** Heat map showing significantly altered lipids (stratified by fatty acid chain length: degree of saturation) in AA PCa vs matched benign patient prostate tissues. Shades of Yellow and Blue represent up and down regulated lipids, respectively (see color key). Lipid classes are marked on the left side of the heatmap. Lipid classes include PE: Phosphatidyl ethanolamine, PC: Phosphatidyl choline, PI: Phosphatidyl inositol, PG: Phosphatidyl Glycerol, PS: Phosphatidyl Serine, L-PE: Lyso Phosphatidyl Ethanolamine, TG: Triglycerides, SM: Sphingomyelin, P-PE: Plasmenyl Phosphatidyl Ethanolamine, CE: Cholesteryl Esters, DG: Diglycerides, L-PC: Lyso phosphatidyl choline. **(D)** Average levels of L-PC (p=0.042) are significantly down-regulated in high Gleason grade (High GL, n=26) compared to Low Gleason grade (Low GL, n=22) tumors. Along similar lines, lower average levels of L-PC (p=0.0079) SM (p=0.036) and PE (p=0.057) are associated with biochemical recurrence (BCR within 5 years post-prostatectomy) in PCa patients. No BCR (n=15); BCR (n=10). For panels B and C, paired t-test followed by Benjamini Hochberg (BH) False Discovery Rate (FDR<0.1) correction was used to compute differential analysis. For panel D, Mann-Whitney test with BH FDR<0.25 was used to compute statistical significance.

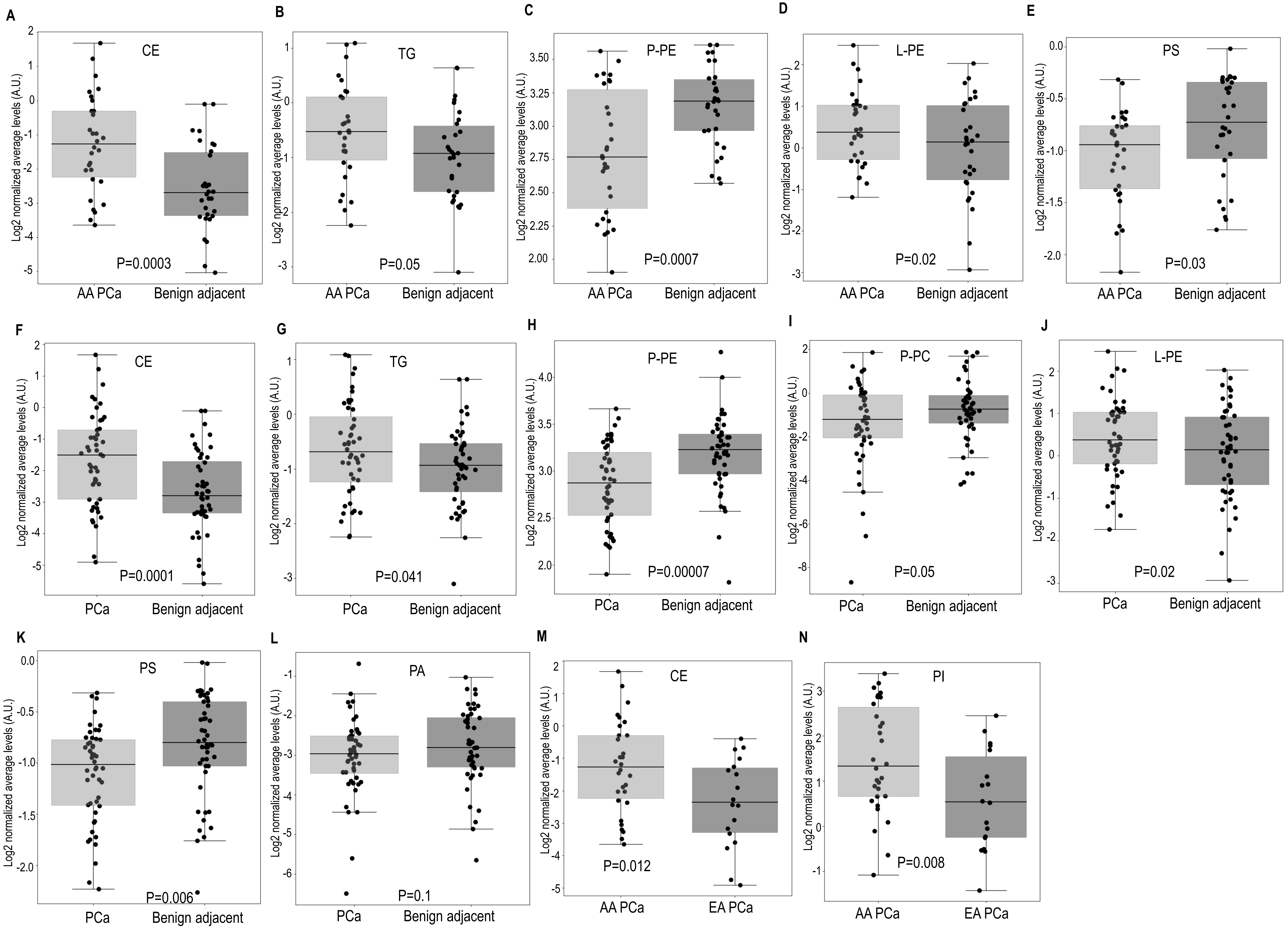

Supplemental Figure S4. Box plots for altered lipid classes. (A-E) AA PCa vs. Benign adjacent (n=30 pairs). **(F-L)** PCa vs Benign adjacent (n=48 pairs). **(M-N)** AA PCa (n=30) vs. EA PCa (n=18). Normalized intensity values for lipids belonging to each class (refer Table 2) were averaged. Mann-Whitney test with BH FDR<0.25 was used to compute statistical significance for each lipid class.
