# Supplementary material for: Lipid Alterations in African American Men with Prostate Cancer
Source: Metabolites. 2021 Dec 22;12(1):8. doi: 10.3390/metabo12010008 (PMC8779756; doi:10.3390/metabo12010008)
Supplement: Supplementary file 1 [file metabolites-12-00008-s001.zip › metabolites-1471570-supplementary/Supplementary Tables.pdf]

Supplementary Table S1. List of lipids that were loaded onto axes on Principal Component Analysis plots for total PCa vs matched benign tissues. List of lipids that were loaded onto PC1 and PC2 axes for comparison of differential lipids in PCa vs matched benign tissues. PE: Phosphatidyl ethanolamine, PC: Phosphatidyl choline, PI: Phosphatidyl inositol, PG: Phosphatidyl Glycerol, PS: Phosphatidyl Serine, L-PE: Lyso-Phosphatidyl Ethanolamine, TG: Triglycerides, SM: Sphingomyelin, P-PE: Plasmenyl-Phosphatidyl Ethanolamine, CE: Cholesteryl Esters, DG: Diglycerides, CL: Cardiolipins, L-PC: Lyso-Phosphatidyl Choline. The fatty acid chain length and the number of bonds (degree of saturation) are indicated.

Loadings	PC1 (23.95%)	PC2 (12.2%)
CE.18.0	-0.11427	-0.02586
CE.19.0	-0.07917	-0.03358
CE.20.0	-0.11912	-0.05028
CE.16.1	-0.08994	-0.0018
CE.18.1	-0.0769	0.006708
CE.20.1	-0.1259	-0.03679
CE.22.1	-0.12999	-0.0458
CE.24.1	-0.10517	-0.05128
CE.22.2	-0.13114	-0.03251
CE.18.3	-0.09304	-0.00118
CE.20.3	-0.11624	-0.01894
CE.22.4	-0.11576	-0.00708
CE.22.5	-0.07287	0.016097
CE.22.6	-0.10797	-0.01114
DG.32.0	0.023041	0.112769
DG.34.0	0.019578	0.087714
DG.36.0	0.014869	0.081953
DG.35.1	-0.11424	0.044818
DG.37.1	-0.10889	0.054645
DG.38.1	-0.11541	0.027693
DG.35.2	-0.08175	0.021206
DG.37.2	-0.11091	0.011199
DG.38.2	-0.11942	0.047716
DG.40.2	-0.11815	0.025742
DG.36.4	-0.02925	0.100023
DG.37.4	-0.01498	0.081405
DG.38.4	0.010849	0.129481
DG.38.5	-0.02733	0.125378
DG.40.5	-0.03983	0.113127
DG.38.6	-0.0432	0.114121
lysoPC.17.0	0.03915	0.103928
lysoPE.16.1	-0.07868	0.020012

lysoPE.22.1	-0.07801	0.023507
lysoPE.18.2	-0.07083	0.020832
lysoPE.20.3	-0.02971	0.019686
lysoPE.22.6	-0.00741	0.024279
PA.37.4	0.029469	0.072295
PC.28.0	-0.08963	0.033385
PC.31.0	0.031524	0.088511
PC.32.0	0.021375	0.126324
PC.33.0	0.01084	0.042357
PC.36.0	-0.07539	0.030791
PC.34.1	-0.0232	0.105885
PC.35.1	-0.06786	0.054337
PC.38.1	-0.05599	0.048552
PC.36.3	0.079252	0.063731
PC.34.4	-0.00184	0.106515
PC.36.4	0.04538	0.134168
PC.38.4	-0.00203	0.100228
PC.40.4	0.066121	0.03626
PC.37.5	-0.01972	0.115472
PC.40.6	-0.00382	0.13576
PC.40.7	0.016755	0.06306
PC.38.8	0.002338	0.120238
PC.40.8	-0.03732	-0.00951
PC.42.9	0.058092	0.067078
PC.40.10	0.005574	0.117152
PE.30.0	-0.01216	0.127506
PE.34.0	0.055174	0.093285
PE.36.1	-0.02797	0.093514
PE.40.1	-0.0651	0.043175
PE.32.2	-0.08189	0.021098
PE.33.2	-0.05002	0.065123
PE.37.2	-0.11307	0.018119
PE.35.3	-0.04562	0.013344
PE.36.3	-0.06521	0.051087
PE.40.3	-0.06718	0.053749
PE.36.4	0.027818	0.068586
PE.37.4	-0.08266	0.054372
PE.38.4	0.023471	0.0338
PE.39.4	-0.08331	0.046962
PE.40.4	-0.00538	0.08394
PE.41.4	-0.05119	0.032558

PE.42.4	-0.06727	0.039846
PE.38.5	0.002993	0.111558
PE.44.5	-0.08407	0.020005
PE.41.6	-0.04174	-0.00511
PE.42.6	-0.06365	0.035542
PE.42.7	-0.08201	0.052063
PG.34.0	0.071474	0.077109
PG.34.1	-0.01072	0.08579
PG.36.1	0.047644	0.051701
PG.36.3	-0.09984	0.056544
PG.38.4	-0.09767	0.039368
PG.38.4.1	0.056544	0.120665
PG.44.11	-0.02732	0.095067
PI.36.2	-0.07543	-0.02934
PI.38.3	-0.05584	-0.0053
PI.40.4	-0.01597	0.014705
plasmenyl.PC.18.0	-0.01917	0.038665
plasmenyl.PE.32.0	0.010675	0.127157
plasmenyl.PE.36.1	-0.01141	0.111943
plasmenyl.PE.36.4	-0.00861	0.158336
plasmenyl.PE.36.4.1	0.003436	0.144229
plasmenyl.PE.38.4	0.057697	0.160316
plasmenyl.PE.40.4	0.034782	0.120224
plasmenyl.PE.42.4	-0.0081	0.101871
plasmenyl.PE.36.5	0.00945	0.068108
plasmenyl.PE.36.5.1	-0.01732	0.115462
plasmenyl.PE.36.5.2	-0.0119	0.121429

Supplementary Table S2: List of lipids that were loaded onto axes on Principal Component Analysis plots for AA PCa vs matched benign tissues. List of lipids that were loaded onto PC1 and PC2 axes for comparison of differential lipids in AA PCa vs matched benign tissues. PE: Phosphatidyl ethanolamine, PC: Phosphatidyl choline, PI: Phosphatidyl inositol, PG: Phosphatidyl Glycerol, PS: Phosphatidyl Serine, L-PE: Lyso-Phosphatidyl Ethanolamine, TG: Triglycerides, SM: Sphingomyelin, P-PE: Plasmenyl-Phosphatidyl Ethanolamine, CE: Cholesteryl Esters, DG: Diglycerides, CL: Cardiolipins, L-PC: Lyso-Phosphatidyl Choline. The fatty acid chain length and the number of bonds (degree of saturation) are indicated.

Loadings	PC1 (29.58%)	PC2 (11.04%)
CE.16.1	-0.07569	0.015076
CE.18.0	-0.1072	0.056362
CE.18.1	-0.1097	0.038086
CE.18.3	-0.07846	0.029549
CE.19.0	-0.06071	0.054927
CE.20.0	-0.10363	0.062265
CE.20.1	-0.12443	0.050558
CE.20.3	-0.10409	0.039714
CE.20.5	-0.06815	-0.02028
CE.22.1	-0.12497	0.056981
CE.22.2	-0.11928	0.054592
CE.22.4	-0.10547	0.033513
CE.22.5	-0.08515	-0.0047
CE.22.6	-0.10372	0.040171
CE.24.1	-0.09379	0.082391
DG.32.0	0.046776	-0.12616
DG.33.0	-0.02524	-0.10766
DG.34.0	0.042004	-0.10916
DG.35.0	-0.06799	-0.05333
DG.35.1	-0.1095	-0.06451
DG.35.2	-0.0877	-0.03269
DG.36.0	0.036786	-0.10508
DG.37.1	-0.10362	-0.05926
DG.37.2	-0.10682	-0.02943
DG.37.4	-0.01635	-0.10677
DG.38.1	-0.10047	-0.03729
DG.38.2	-0.10506	-0.05666
DG.38.4	0.011728	-0.12875
DG.38.5	-0.02188	-0.11858
DG.40.2	-0.10363	-0.04189
DG.40.4	-0.02637	-0.10395
DG.40.5	-0.03722	-0.11871

lysoPC.17.0	0.054534	-0.12941
lysoPE.16.1	-0.07542	0.008252
lysoPE.17.0	-0.07574	-0.01651
lysoPE.18.0	-0.06467	-0.02714
lysoPE.18.1	-0.08023	-0.01712
lysoPE.18.2	-0.06796	0.002531
lysoPE.19.0	-0.06682	-0.01633
lysoPE.20.0	-0.0661	0.000522
lysoPE.22.1	-0.08478	-0.00273
PC.28.0	-0.08442	-0.0297
PC.31.0	0.025325	-0.09397
PC.32.0	0.005693	-0.11004
PC.33.0	0.025459	-0.07382
PC.35.1	-0.06984	-0.06675
PC.36.0	-0.07387	-0.04362
PC.36.3	0.085364	-0.08567
PC.36.4	0.03567	-0.12324
PC.37.6	-0.07735	-0.08789
PC.38.1	-0.05548	-0.05565
PC.38.6	-0.0725	-0.02768
PC.40.6	-0.00368	-0.1341
PC.40.7	0.019639	-0.07295
PC.40.8	-0.04836	0.000415
PC.42.10	-0.06996	0.00525
PC.42.6	-0.0295	0.039781
PC.42.9	0.056231	-0.07932
PE.30.0	-0.01419	-0.14256
PE.32.2	-0.08751	-0.02016
PE.34.0	0.070235	-0.11191
PE.36.1	-0.01968	-0.14382
PE.36.3	-0.05889	-0.04698
PE.36.4	0.033528	-0.09679
PE.37.2	-0.11179	-0.00755
PE.37.4	-0.09057	-0.02344
PE.38.4	0.039145	-0.0685
PE.39.7	-0.08601	-0.06674
PE.40.1	-0.06476	-0.04506
PE.40.4	-0.00652	-0.10419
PE.40.8	-0.07108	-0.04476
PE.41.4	-0.05571	0.005634
PE.41.6	-0.03901	0.011053

PE.42.4	-0.07483	-0.01813
PE.42.6	-0.07204	-0.01699
PE.42.7	-0.08892	-0.03573
PE.44.4	-0.07822	-0.03059
PE.44.5	-0.09128	0.003774
PG.34.0	0.073537	-0.0913
PG.36.1	0.041701	-0.04809
PG.36.2	-0.10487	-0.01293
PG.36.3	-0.10596	-0.02962
PG.38.3	-0.08787	-0.05198
PG.38.4	-0.10435	-0.02365
PG.38.4	0.048484	-0.12873
PG.38.5	-0.09105	-0.05118
PG.42.6	-0.06836	-0.08248
PI.36.2	-0.07542	0.050037
PI.38.3	-0.06622	0.018844
PI.39.4	-0.05193	0.040756
plasmenyl.PE.32.0	0.015773	-0.14748
plasmenyl.PE.36.1	0.005009	-0.15074
plasmenyl.PE.36.4	0.001648	-0.16317
plasmenyl.PE.36.4	0.010171	-0.13976
plasmenyl.PE.36.5	0.001315	-0.07447
plasmenyl.PE.36.5	-0.01903	-0.1086
plasmenyl.PE.36.5	-0.01274	-0.09693
plasmenyl.PE.38.4	0.060073	-0.16023
plasmenyl.PE.38.5	0.022827	-0.16944
plasmenyl.PE.38.5	0.038443	-0.15094

Supplementary Table S3. List of differential lipids (FDR<0.10) in AA PCa vs matched benign adjacent tissues. List of detected differential lipids between AA PCa and matched benign adjacent tissue arranged by lipid name, class, length, and bond number. PE: Phosphatidyl ethanolamine, PC: Phosphatidyl choline, PI: Phosphatidyl inositol, PG: Phosphatidyl Glycerol, PS: Phosphatidyl Serine, L-PE: Lyso-Phosphatidyl Ethanolamine, TG: Triglycerides, SM: Sphingomyelin, P-PE: Plasmenyl-Phosphatidyl Ethanolamine, CE: Cholesteryl Esters, DG: Diglycerides, CL: Cardiolipins, L-PC: Lyso-Phosphatidyl Choline. The fatty acid chain length and the number of bonds (degree of saturation) are indicated.

Lipid Name	Length of chain	Bond Number
CE 16:1; [M+NH4]+@10.16	16	1
CE 18:0; [M+NH4]+@10.84	18	0
CE 18:1; [M+NH4]+@10.51	18	1
CE 18:3; [M+NH4]+@10.03	18	3
CE 19:0; [M+NH4]+@11.00	19	0
CE 20:0; [M+NH4]+@11.12	20	0
CE 20:1; [M+NH4]+@10.85	20	1
CE 20:3; [M+NH4]+@10.30	20	3
CE 22:1; [M+NH4]+@11.23	22	1
CE 22:2; [M+NH4]+@10.87	22	2
CE 22:4; [M+NH4]+@10.40	22	4
CE 22:5; [M+NH4]+@10.22	22	5
CE 22:6; [M+NH4]+@10.00	22	6
CE 24:1; [M+NH4]+@11.39	24	1
DG 32:0; [M+NH4]+@7.12	32	0
DG 34:0; [M+NH4]+@7.66	34	0
DG 36:0; [M+NH4]+@8.13	36	0
DG 37:2; [M+NH4]+@7.64	37	2
DG 38:4; [M+NH4]+@7.17	38	4
lysoPC 17:0; [M+H]+@2.40	17	0
lysoPE 16:1; [M-H]-@1.19	16	1
lysoPE 18:2; [M-H]-@1.22	18	2
lysoPE 20:0; [M-H]-@3.44	20	0
lysoPE 22:1; [M+H]+@2.55	22	1
PC 35:1; [M-Ac-H]-@7.07	35	1
PC 36:3; [M+H]+@7.06	36	3
PC 36:4; [M+H]+@5.835	36	4
PC 40:7; [M+H]+@9.10	40	7
PC 42:9; [M+H]+@6.13	42	9
PE 30:0; [M+H]+@5.40	30	0
PE 34:0; [M+Na]+@6.695	34	0
PE 36:1; [M+Na]+@6.77	36	1
PE 36:4; [M-H]-@7.66	36	4
PE 37:2; [M-H]-@7.28	37	2
PE 38:4; [M-H]-@7.86	38	4

PE 40:4; [M+H]+@6.94	40	4
PE 41:6; [M-H]-@7.21	41	6
PE 42:4; [M-H]-@7.94	42	4
PE 42:6; [M-H]-@7.46	42	6
PE 42:7; [M-H]-@6.96	42	7
PE 44:5; [M-H]-@8.09	44	5
PG 34:0; [M-H]-@6.295	34	0
PG 36:1; [M-H]-@6.45	36	1
PG 36:2; [M-H]-@5.85	36	2
PG 36:3; [M-H]-@5.37	36	3
PG 38:3; [M-H]-@5.81	38	3
PG 38:4; [M-H]-@5.44	38	4
PG 38:4; [M-H]-@6.09	38	4
PI 36:2; [M-H]-@5.90	36	2
PI 38:3; [M-H]-@6.13	38	3
PI 39:4; [M-H]-@6.23	39	4
plasmenyl-PE 32:0; [M+H]+@6.41	32	0
plasmenyl-PE 36:1; [M+Na]+@7.075	36	1
plasmenyl-PE 36:4; [M+H]+@5.85	36	4
plasmenyl-PE 36:4; [M-H]-@6.67	36	4
plasmenyl-PE 36:5; [M+H]+@4.04	36	5
plasmenyl-PE 36:5; [M-H]-@6.35	36	5
plasmenyl-PE 38:4; [M+Na]+@6.875	38	4
plasmenyl-PE 38:5; [M+H]+@5.91	38	5
plasmenyl-PE 38:5; [M-H]-@6.76	38	5
plasmenyl-PE 38:6; [M+H]+@5.77	38	6
plasmenyl-PE 38:6; [M-H]-@6.60	38	6
plasmenyl-PE 40:4; [M+Na]+@7.3625	40	4
plasmenyl-PE 40:5; [M+H]+@6.63	40	5
plasmenyl-PE 40:5; [M-H]-@7.47	40	5
plasmenyl-PE 42:4; [M-H]-@8.09	42	4
PS 36:2; [M+Na]+@4.94	36	2
PS 36:4; [M-H]-@5.32	36	4
PS 38:4; [M+Na]+@4.92	38	4
PS 38:5; [M-H]-@5.47	38	5
PS 38:6; [M-H]-@5.22	38	6
PS 38:7; [M+H]+@4.38	38	7
PS 40:4; [M-H]-@6.40	40	4
PS 40:6; [M+Na]+@4.78	40	6
SM 30:1; [M]+@3.51	30	1
SM 35:1; [M+Na]+@8.43	35	1
SM 35:2; [M]+@5.07	35	2
SM 41:4; [M]+@6.18	41	4

SM 41:5; [M]+@6.10	41	5
SM 42:2; [M]+@9.01	42	2
TG 40:0; [M+NH4]+@8.83	40	0
TG 46:0; [M+NH4]+@9.785	46	0
TG 50:0; [M+NH4]+@10.33	50	0
TG 51:1; [M+NH4]+@10.225	51	1
TG 52:1; [M+NH4]+@10.325	52	1
TG 53:0; [M+NH4]+@10.685	53	0
TG 53:2; [M+NH4]+@10.25	53	2
TG 54:1; [M+NH4]+@10.6	54	1
TG 54:2; [M+NH4]+@10.365	54	2
TG 55:1; [M+NH4]+@10.77	55	1
TG 55:3; [M+NH4]+@10.31	55	3
TG 56:0; [M+NH4]+@11.035	56	0
TG 56:1; [M+NH4]+@10.82	56	1
TG 56:2; [M+NH4]+@10.62	56	2
TG 56:3; [M+NH4]+@10.4	56	3
TG 58:1; [M+NH4]+@11.06	58	1
TG 58:2; [M+NH4]+@10.82	58	2
TG 58:3; [M+NH4]+@10.63	58	3
TG 58:4; [M+NH4]+@10.44	58	4
TG 58:5; [M+NH4]+@10.295	58	5
TG 60:1; [M+NH4]+@11.26	60	1
TG 60:2; [M+NH4]+@11.06	60	2
TG 60:3; [M+NH4]+@10.85	60	3
TG 60:4; [M+NH4]+@10.67	60	4
TG 60:6; [M+NH4]+@10.34	60	6

Supplementary Table S4. List of differential lipids (FDR<0.10) in PCa vs adjacent benign tissues. List of detected differential lipids between PCa and matched benign adjacent tissue arranged by lipid name, length, and bond number. PE: Phosphatidyl ethanolamine, PC: Phosphatidyl choline, PI: Phosphatidyl inositol, PG: Phosphatidyl Glycerol, PS: Phosphatidyl Serine, L-PE: Lyso-Phosphatidyl Ethanolamine, TG: Triglycerides, SM: Sphingomyelin, P-PE: Plasmenyl-Phosphatidyl Ethanolamine, CE: Cholesteryl Esters, DG: Diglycerides, CL: Cardiolipins, L-PC: Lyso-Phosphatidyl Choline. The fatty acid chain length and the number of bonds (degree of saturation) are indicated.

Lipid Name	Length of chain	Bond Number
CE 16:1	16	1
CE 18:0	18	0
CE 18:3	18	3
CE 19:0	19	0
CE 20:0	20	0
CE 20:1	20	1
CE 20:3	20	3
CE 22:1	22	1
CE 22:2	22	2
CE 22:4	22	4
CE 22:6	22	6
CE 24:1	24	1
DG 32:0	32	0
DG 34:0	34	0
DG 35:1	35	1
DG 35:2	35	2
DG 36:0	36	0
DG 36:4	36	4
DG 37:2	37	2
DG 37:4	37	4
DG 38:1	38	1
DG 38:2	38	2
DG 38:4	38	4
DG 38:5	38	5
DG 40:2	40	2
lysoPC 17:0	17	0
lysoPE 16:1	16	1
lysoPE 18:2	18	2
lysoPE 22:1	22	1
PA 37:4	37	4
PC 28:0	28	0
PC 31:0	31	0
PC 32:0	32	0
PC 35:1	35	1
PC 36:3	36	3

PC 36:4	36	4
PC 40:6	40	6
PC 40:7	40	7
PC 42:9	42	9
PE 30:0	30	0
PE 34:0	34	0
PE 36:1	36	1
PE 36:4	36	4
PE 37:2	37	2
PE 38:4	38	4
PE 38:5	38	5
PE 40:1	40	1
PE 40:3	40	3
PE 40:4	40	4
PE 41:4	41	4
PE 41:6	41	6
PE 42:4	42	4
PE 42:6	42	6
PE 44:5	44	5
PG 34:0	34	0
PG 36:1	36	1
PG 38:4	38	4
PI 36:2	36	2
plasmenyl-PE 32:0	32	0
plasmenyl-PE 36:4	36	4
plasmenyl-PE 36:4	36	4
plasmenyl-PE 36:5	36	5
plasmenyl-PE 36:5	36	5
plasmenyl-PE 38:4	38	4
plasmenyl-PE 38:5	38	5
plasmenyl-PE 38:5	38	5
plasmenyl-PE 38:6	38	6
plasmenyl-PE 40:4	40	4
plasmenyl-PE 40:5	40	5
plasmenyl-PE 40:5	40	5
plasmenyl-PE 42:4	42	4
PS 36:4	36	4
PS 38:4	38	4
PS 38:5	38	5
PS 38:6	38	6
PS 38:7	38	7
PS 40:4	40	4
PS 40:6	40	6

SM 30:1	30	1
SM 35:1	35	1
SM 35:2	35	2
SM 41:4	41	4
SM 41:5	41	5
SM 42:2	42	2
TG 40:0	40	0
TG 42:0	42	0
TG 46:0	46	0
TG 48:0	48	0
TG 48:1	48	1
TG 49:0	49	0
TG 49:1	49	1
TG 50:0	50	0
TG 50:1	50	1
TG 51:1	51	1
TG 52:0	52	0
TG 52:1	52	1
TG 53:0	53	0
TG 53:2	53	2
TG 54:1	54	1
TG 54:2	54	2
TG 55:3	55	3
TG 56:0	56	0
TG 56:1	56	1
TG 56:2	56	2
TG 56:3	56	3
TG 58:1	58	1
TG 58:2	58	2
TG 58:3	58	3
TG 58:4	58	4
TG 58:5	58	5
TG 60:1	60	1
TG 60:2	60	2
TG 60:3	60	3
TG 60:4	60	4
TG 60:6	60	6

Supplementary Table S5: List of lipids that were loaded onto axes on Principal Component Analysis plots for AA PCa vs EA PCa tissues. List of lipids that were loaded onto PC1 and PC2 axes for comparison of differential lipids in AA PCa vs EA PCa tissues. PE: Phosphatidyl ethanolamine, PC: Phosphatidyl choline, PI: Phosphatidyl inositol, PG: Phosphatidyl Glycerol, PS: Phosphatidyl Serine, L-PE: Lyso-Phosphatidyl Ethanolamine, TG: Triglycerides, SM: Sphingomyelin, P-PE: Plasmenyl-Phosphatidyl Ethanolamine, CE: Cholesteryl Esters, DG: Diglycerides, CL: Cardiolipins, L-PC: Lyso-Phosphatidyl Choline. The fatty acid chain length and the number of bonds (degree of saturation) are indicated.

Loadings	PC1 (30.63%)	PC2 (14.56%)
CE.18.0	0.222137	-0.14586
CE.19.0	0.167288	-0.20917
CE.20.0	0.241701	-0.16705
CE.16.1	0.17609	-0.19915
CE.18.1	0.194858	-0.03422
CE.20.1	0.237355	-0.11595
CE.22.1	0.25193	-0.1484
CE.22.2	0.237251	-0.1472
CE.20.5	0.162516	-0.20595
CE.22.5	0.158068	-0.06642
CE.22.6	0.208212	-0.18259
PA.34.0	-0.10447	-0.10485
PA.37.4	0.055306	0.152355
PC.32.0	-0.15902	0.083578
PC.42.10	0.193075	0.240151
PG.36.2	0.214621	0.113058
PI.36.2	0.198271	0.241624
PI.38.3	0.088546	0.270868
PI.36.4	0.157119	0.291103
PI.38.4	0.16105	0.285351
PI.39.4	0.154679	0.187326
PI.40.4	0.146932	0.081324
plasmenyl.PC.20.0	-0.03051	0.060882
plasmenyl.PE.32.0	-0.11715	0.102713
SM.43.1	-0.06949	0.170154
SM.43.1.1	-0.03051	0.045271
SM.44.1	-0.10136	0.187693
SM.41.4	-0.15503	-0.03239
TG.55.1	0.194951	-0.10912
TG.60.5	0.214617	-0.0806

Supplementary Table S6. List of differential lipids (FDR<0.10) comparing AA vs EA PCa tumors. List of detected differential lipids between AA vs EA PCa tissues arranged by lipid name, length, and bond number. PE: Phosphatidyl ethanolamine, PC: Phosphatidyl choline, PI: Phosphatidyl inositol, PG: Phosphatidyl Glycerol, PS: Phosphatidyl Serine, L-PE: Lyso-Phosphatidyl Ethanolamine, TG: Triglycerides, SM: Sphingomyelin, P-PE: Plasmenyl-Phosphatidyl Ethanolamine, CE: Cholesteryl Esters, DG: Diglycerides, CL: Cardiolipins, L-PC: Lyso-Phosphatidyl Choline. The fatty acid chain length and the number of bonds (degree of saturation) are indicated.

Lipid Name	Length of chain	Bond Number
CE 18:0	18	0
CE 18:1	18	1
CE 20:0	20	0
CE 20:1	20	1
CE 22:1	22	1
CE 22:2	22	2
PG 36:2	36	2
PI 36:2	36	2
PI 38:4	38	4
PI 39:4	39	4
SM 41:4	41	4
TG 55:1	55	1
